# Lifetime body size and estrogen-receptor-positive breast cancer risk in the California Teachers Study cohort

**DOI:** 10.1186/s13058-016-0790-5

**Published:** 2016-12-21

**Authors:** Pamela L. Horn-Ross, Alison J. Canchola, Leslie Bernstein, Susan L. Neuhausen, David O. Nelson, Peggy Reynolds

**Affiliations:** 1 Cancer Prevention Institute of California, 2201 Walnut Ave, Suite 300, Fremont, CA 94538 USA; 2 Cancer Prevention Institute of California, 2001 Center St, Suite 700, Berkeley, CA 94704 USA; 3 Department of Population Sciences, Beckman Research Institute, City of Hope, 1500 Duarte Rd, Duarte, CA 91010 USA; 4 Division of Epidemiology, Department of Health Research and Policy, Stanford University School of Medicine, 150 Governor’s Lane, Stanford, CA 94305 USA

**Keywords:** Breast cancer, Body size, Obesity, Adiposity, Body fat distribution, Height, Life course

## Abstract

**Background:**

Obesity is a public health epidemic and an important breast cancer risk factor. The relationship between interrelated body measurements is complex and most studies fail to account for this complexity. We identified key aspects of body size which jointly, over the life-course (since adolescence), are associated with estrogen-receptor-positive (ER+) breast cancer risk.

**Methods:**

Among 109,862 women participating in the California Teachers Study cohort, 3844 were diagnosed with invasive ER+ breast cancer between 1997–1998 and December 2011. Based on validated self-reported height and weight at age 18, baseline, and 10-year follow up and waist circumference at 2-year and 10-year follow up, we identified 16 *a priori* body-size phenotypes. Multivariable Cox proportional hazards models provided estimates of hazard rate ratios (HR) and 95% confidence intervals (CI).

**Results:**

Premenopausal breast cancer was influenced by adolescent, but not adult, body size (HR = 0.51, 95% CI 0.31–0.86 for body mass index (BMI; kg/m^2^) ≥25 vs <20 at age 18). Among postmenopausal women currently using hormone therapy, only those with the greatest body size had increased breast cancer risk (HR = 1.36, 95% CI 1.13–1.64 for height ≥67 inches and adult BMI ≥25 vs height <67). Among postmenopausal women not currently using hormone therapy, the relationship between body size and risk was complex, with the largest effects of adiposity among short women. Among short women, those with gluteal adiposity (HR = 2.70, 95% CI 1.77–4.10) and those who continued to gain weight throughout adulthood (HR = 2.57, 95% CI 1.60–4.12) were at greatest risk, whereas those who had been overweight/obese since adolescence were not at increased risk (HR = 1.33, 95% CI 0.84–2.10). Height was associated with a small increased risk, with borderline statistical significance.

**Conclusions:**

Considering absolute body mass in adolescence and at two points in adulthood, dynamic changes in adiposity over time, and body fat distribution, we identified obesity phenotypes associated with ER+ breast cancer risk. Our approach more clearly identifies specific risk groups than do analyses that evaluate similar measures separately. These findings may aid in improving risk prediction models and developing targeted interventions, and may clarify inconsistent findings across studies.

## Background

One of the most serious public health crises of the last 30 years worldwide has been the rapidly increasing prevalence of overweight and obese individuals and the impact of this epidemic on the risk of many cancers and other chronic diseases. It is well-established that body size impacts breast cancer risk and that these associations vary by menopausal status and the use of hormone therapy (HT) [1–3]. The risk of premenopausal breast cancer is elevated among tall women and reduced among obese women [1, 2]. Overall, postmenopausal women are at greater risk of breast cancer if they are tall or obese or have substantial adult weight gain or abdominal adiposity [1, 2]. However, these effects are limited to women who have not used HT [1, 2, 4–7]. Thus, with the substantial reduction in HT use since the early 2000s, body size may have a greater impact on postmenopausal breast cancer risk than in past decades. Both risk and the mechanisms involved also vary by the hormone receptor status of the tumor, particularly whether it is estrogen-responsive [3, 8].

Most epidemiologic studies have examined body size measures as single independent variables or as the joint effects of two variables. However, the interrelationships between various body measurements are complex - individual measures are often correlated with each other (e.g., weight and waist circumference) and over time within an individual. In addition, the associations between body size and disease risk reflect confounding and interactions and can vary over the life-course. For example, obesity early in life may not only reduce the risk of premenopausal breast cancer, but continued obesity throughout life may delay or ameliorate the risk of postmenopausal breast cancer normally associated with obesity in later life [4, 9, 10]. Thus, our objective was to identify key aspects of body size over the life-course (since adolescence) that are associated with risk of the most commonly diagnosed breast cancers (i.e., those that are estrogen-receptor-positive (ER+)), jointly considering aspects of absolute obesity and stature, changes in adiposity over time, and body fat distributions using data from our large, diverse cohort of women prospectively followed for more than 10 years.

## Methods

The California Teachers Study (CTS) recruited 133,479 active and retired female public school teachers, administrators, and other professionals in 1995–1996 [11]. At the time participants joined the cohort (baseline), they completed a self-administered questionnaire addressing health and medical history (including menopausal status, HT use, and body size), lifestyle, and other exposures and behaviors. The second follow-up questionnaire, completed in 1997–1998, included self-measured waist and hip circumferences. The third (2000–2001) updated menopausal status and HT use and the fourth (2005–2006) updated menopausal status, HT use, and body size measures. Response rates to the follow-up questionnaires were 75%, 74%, and 67%, respectively, with 88% of the active cohort completing one or more follow-up questionnaires. The CTS was approved by the Institutional Review Boards at the Cancer Prevention Institute of California, City of Hope National Medical Center, the University of Southern California, the University of California at Irvine, and the California Health and Human Services Agency.

### Assessment of body size

Body size measures included in the present analysis were height, body mass index (BMI; kg/m^2^), weight change, and waist-to-height ratio (WHtR). Self-reported height (in inches) at baseline and at age 18 years were highly correlated (*r* = 0.97). Thus, adult height was defined as height at baseline and if missing, as height at age 18. BMI was calculated based on self-reported weight (reported in pounds and converted to kilograms) at age 18 and baseline and adult height as described above (converted to meters). At the 2-year follow up, women were provided a standard heavy-weight flexible paper tape measure (calibrated in inches on both sides with each side a different color to prevent errors in measurement) and asked participants to measure their waist and hip circumferences following written, illustrated instructions. We chose to use WHtR, instead of waist-to-hip ratio, as a measure of abdominal adiposity as the former has been found to be a better predictor of visceral fat, while the latter is more strongly correlated with subcutaneous fat [12, 13]. Details on the measurement and validation of body measurements have been published elsewhere [4]. The 10-year follow-up questionnaire included questions to update information on weight and waist circumference. Weight change between age 18 and baseline and baseline and the 10-year follow-up were calculated.

We used the following measures to identify lifetime body size phenotypes: adult height; adolescent BMI (at age 18 years); BMI at baseline; weight change from age 18 to baseline among women of normal weight (i.e., BMI <25) at baseline; and WHtR among women who were overweight (i.e., BMI 25.0–29.9) or obese (BMI ≥30) at baseline. Based on *a priori* decisions, we evaluated weight change only among women of normal weight (BMI <25) under the assumption that among overweight/obese women the BMI attained was more important than the amount of weight gain. Similarly, body fat distribution (WHtR) was evaluated only among overweight/obese women under the assumption that in the absence of significant adipose tissue among women of normal weight, the site of that tissue had minimal effect. These assumptions also kept the total number of phenotypes evaluated at a reasonable number and were further justified by the very small number of women in most of the subgroups when additional stratification as described was examined post hoc. In a second set of analyses we also incorporated BMI at the 10-year follow up and weight change from baseline to the 10-year follow up, the latter among women of normal weight at the 10-year follow up.

### Follow up for outcomes

The CTS cohort is followed annually for cancer diagnoses, changes of address, and deaths. Cancer diagnoses are determined by linkage with the California Cancer Registry (CCR), a population-based cancer registry, which contributes data to the National Cancer Institute Surveillance, Epidemiology, and End Results program, and covers the entire state of California. As more than 99% of all cancer diagnoses among California residents are reported to the CCR [14], cohort members who continue to reside in California are effectively in active follow up of cancer outcomes. Changes of address are obtained by annual mailings, notifications from participants, and record linkages with multiple sources including the US Postal Service National Change of Address database. California and national mortality files are used to ascertain date of death.

### Study population

For the present analysis, we sequentially excluded women, who at baseline were judged to have invalid data overall (n = 2); did not live in California (n = 8867); had a prior history of breast cancer (identified by self-report or by linkage with the CCR; n = 6212) or whose prior history of cancer was unknown (n = 139); had a bilateral mastectomy prior to joining the cohort (n = 11); or were aged 85 years or older (n = 1980). We began follow up for the present analysis at the time that the 2-year follow-up questionnaire was completed (because this was when abdominal adiposity was assessed). Thus, we also excluded women who had moved out of California (n = 1711), died (n = 674), or had been diagnosed with an invasive or *in-situ* breast cancer during that time period (n = 754 and 151, respectively). Also excluded were 530 women with breast cancer whose ER status was unknown and 2586 women with unknown menopausal status or HT data at all three assessments (i.e., baseline, 5-year, and 10-year follow-up). Thus, the analysis cohort included 109,862 women among whom 3844 were diagnosed with incident invasive ER+ breast cancer between completing the 2-year follow up in 1997–1998 and 31 December 2011.

### Statistical analysis

Multivariable Cox proportional hazards regression was used to estimate hazard rate ratios (HR) and 95% confidence intervals (CI) relating breast cancer to measures of body size over time as a function of menopausal status and current HT use. Follow-up time was calculated as the number of days between completion of the 2-year follow-up questionnaire (or for those not completing this second questionnaire, 5 November 1997, the median date the questionnaire was completed by those who filled it out) and the first of the following six events: diagnosis of invasive ER+ breast cancer (n = 3844), diagnosis of ER-negative (ER-) or *in-situ* breast cancer (n = 684 and 1249, respectively), a move outside of California lasting more than 4 months (n = 9903), death (n = 11,433), a bilateral mastectomy (n = 15), or the end of follow up (31 December 2011).

Menopausal status and HT use were collected at baseline and updated based on data collected at the 5-year and 10-year follow up. For each woman, these characteristics were modeled as time-varying covariates with four levels: premenopausal; postmenopausal, currently using HT; postmenopausal, not using HT; and unknown. Perimenopausal women (those with a last menses within the past six months) were included with the postmenopausal groups based on HT use. The data were represented as a counting process, with transitions at the dates of the two follow-up questionnaires (or the median completion date for non-responders) for each subject whose status changed. Three subsets of the resulting counting process data were created for subsequent analyses (premenopausal; postmenopausal, currently using HT; and postmenopausal, not using HT) and separate regression analyses were performed on each subset. There were 46,847 women who contributed time to the premenopausal subset, 49,581 to the postmenopausal/current HT use subset, and 60,278 women to the postmenopausal/no HT subset.

Age (in days) was used as the time metric in all regression models, with stratification by age (in years) at baseline to adjust for calendar effects. Potential confounders were identified based on prior knowledge and their independent associations with breast cancer risk within each menopausal/HT subset in our cohort. All potential confounders were evaluated in each subset separately. For premenopausal breast cancer, covariates included in the final analyses were a history of benign breast disease (yes, no) and family history of breast cancer in a first-degree relative (yes, no). For postmenopausal breast cancer among women currently using HT, the covariates were nulliparity and among parous women, age at first full-term pregnancy (in years); history of benign breast disease; family history of breast cancer in a first-degree relative; average alcohol consumption in the year prior to baseline (non-drinker, <20 g/day, ≥20 g/day); and neighborhood socioeconomic status (in deciles). For postmenopausal breast cancer among women not using HT covariates were age at menarche (in years from ≤9 to ≥17); nulliparity and among parous women, age at first full-term pregnancy; history of benign breast disease; family history of breast cancer in a first-degree relative; and consumption of a plant-based diet (factor score [15]). Neither physical activity nor four other dietary patterns [15] were associated with risk in any of the menopausal/HT-use groups and only 5% of the women were smokers at baseline [11], thus, these factors were not included as potential confounders.

#### Model selection

For each menopausal/HT subset, our approach for identifying the lifetime body size phenotypes of importance proceeded in three steps. First, we examined the effects of each body size variable separately across a range of levels, collapsing each variable, when possible, into two or three categories based on the observed HRs and CIs, with the goal of maintaining prediction while achieving the most parsimonious model. Second, we used the categories identified from step one to create and evaluate a full model obtained by partitioning the dataset into a set of disjoint phenotype categories based on the joint consideration of height, adolescent BMI, BMI at baseline, weight change among those with normal BMI at baseline, and type of adiposity (abdominal vs gluteal) among those who were overweight or obese, as described above. Finally, we repeatedly collapsed categories created in step two to achieve a final parsimonious model that maximized prediction. Collapsing of categories was based on sample size (precision) within each phenotype and comparisons of between-phenotype differences using the Wald test.

Women with missing data on the covariates included in a given subset were omitted from the analyses reported in Tables 3 and 4. Women with missing data on any of the body size measures needed to define a specific phenotypic category in a given model were included in a single “missing” category for that model. As categories were collapsed, women could reenter the analyses, hence the number of cases reported in the two tables may not exactly sum over the collapsed categories. Once we obtained the best fitting model for each menopausal/HT subset, we further evaluated the effects of later adult body size using the same procedures and BMI and weight change data from the 10-year follow up.

## Results

At the start of follow up, 46,822 women were premenopausal, 36,977 were postmenopausal and currently using HT, and 21,788 were postmenopausal and not using HT (Table 1). Premenopausal women were more likely to be nulliparous than postmenopausal women. Postmenopausal women currently using HT were younger than those who were not using HT. Among parous women, those who were premenopausal were older at the delivery of their first child (median = 28 years) than were women who were postmenopausal (median = 25 years). Premenopausal women were less likely to have had a benign breast biopsy, a family history of breast cancer, consume a plant-based diet, or have more than one alcoholic drink (of around 20 g) per day. HT users were of a slightly higher socioeconomic status than non-users.

**Table 1:** Baseline characteristics of the analytic cohort

	Premenopausal				Postmenopausal, current HT use				Postmenopausal, not using HT			
	(*N* = 46,822)				(*N* = 36,977)				(*N* = 21,788)			
	Number	%	Median	IQR	Number	%	Median	IQR	Number	%	Median	IQR
Age (years)			41	34–46			57	52–65			64	55–72
Age at menarche (years)			12	12–13			12	12–13			13	12–13
Parous	30,580	65.3%			28,793	77.9%			16,440	75.5%		
Age at first full-term pregnancy (among parous)			28	25–30			25	22–28			25	23–29
History of benign breast biopsy	4491	9.6%			7726	20.9%			3776	17.3%		
Mother or sister with breast cancer	4482	9.6%			4503	12.2%			3241	14.9%		
Consumption of a plant-based diet (factor score)			-0.35	-0.86 -0.33			-0.07	-0.65 -0.65			0.01	-0.60 -0.76
Alcohol consumption (g/day)												
none	15,215	32.5%			10,463	28.3%			7810	35.8%		
<20	26,397	56.4%			21,315	57.6%			10,803	49.6%		
≥20	2692	5.7%			3575	9.7%			1918	8.8%		
Socioeconomic status (deciles)												
1–2 (low)	1820	3.9%			1053	2.8%			968	4.4%		
3–4	5858	12.5%			3676	9.9%			2833	13.0%		
5–6	8656	18.5%			5552	15.0%			3683	16.9%		
7–8	14,884	31.8%			10,673	28.9%			6224	28.6%		
9–10 (high)	15,041	32.1%			15,559	42.1%			7777	35.7%		

Percent of missing data: age 0%; age at menarche 1%; parous 2%; age at first full-term pregnancy (n = 2); history of benign breast biopsy 0.4%; mother or sister with breast cancer 3% (includes women who were adopted); dietary factor score 9%; alcohol consumption 5%; and socioeconomic status 1%. *HT* hormone therapy

The most parsimonious version of each of the variables of interest which, when considered separately, predicted ER+ breast cancer risk in each of the menopausal/HT groups is shown in Table 2. A more detailed analysis of these individual body size variables and postmenopausal breast cancer risk in the CTS has been published previously [4]. Adolescent BMI was inversely related to premenopausal breast cancer (HR = 0.51, 95% CI 0.31–0.86 for BMI ≥25 vs BMI <20; *p* trend = 0.006). Adult height was positively associated with risk of postmenopausal breast cancer (HR = 1.19, 95% CI 1.05–1.36 among HT users, and HR = 1.20, 95% CI 1.06–1.35 among women not using HT). Breast cancer among postmenopausal women not using HT was also significantly associated with BMI at baseline and at the 10-year follow up and weight change in both early (age 18 years to baseline) and later (baseline to 10-year follow up) adulthood. The association between risk of breast cancer and abdominal (WHtR ≥0.50), as opposed to gluteal (WHtR <0.50), adiposity approached statistical significance.

**Table 2:** Individual associations between body size and estrogen-receptor-positive breast cancer risk, by menopausal status and HT use

	Premenopausal				Postmenopausal, current HT use				Postmenopausal, not using HT			
	Cases	Person-years	HR^a^	95% CI	Cases	Person-years	HR^b^	95% CI	Cases	Person-years	HR^c^	95% CI
Height (in)												
<65	104	96,093	1.0		887	217,680	1.0		484	170,727	1.0	
65–66	144	124,035	1.10	0.86–1.42					570	178,120	1.20	1.06–1.35
≥67					328	68,919	1.19	1.05–1.36				
missing	0	289			4	561			2	1037		
BMI at age 18 (kg/m^2^)												
<20	99	70,997	1.0		1081	251,998	1.0		883	299,216	1.0	
20–24	127	119,627	0.77	0.59 – 1.00								
≥25	17	24,989	0.51	0.31 – 0.86	88	25,018	0.87	0.70–1.08	114	35,847	1.10	0.90–1.34
missing	5	4805			50	10,144			59	14,821		
BMI at baseline (kg/m2)												
<25	168	150,402	1.0		707	170,986	1.0		523	194,969	1.0	
≥25	75	66,602	0.94	0.71–1.24	469	107,832	1.07	0.95–1.21	488	142,483	1.21	1.07–1.37
missing	5	3414			43	8342			45	12,432		
Weight change (lbs) (age 18 to baseline)												
loss ≥10	163	152,687	1.0		129	29,027	1.16	0.94–1.44	108	36,401	1.35	1.05–1.73
stable					224	56,783	1.0		148	67,402	1.0	
gain 10–24					811	191,026	1.11	0.95–1.28	744	231,034	1.42	1.19–1.70
gain ≥25	79	63,066	1.04	0.79–1.37								
missing	6	4664			55	10,325			56	15,048		
Waist-to-height ratio												
<0.50	127	108,707	1.0		769	180,564	1.0		379	143,507	1.0	
0.50–0.55	52	44,642	0.92	0.67–1.28					465	135,183	1.15	1.00–1.33
≥0.56					229	48,056	1.09	0.94–1.27				
missing	69	67,068			221	58,541			212	71,195		
BMI at 10-year follow up												
<25					140	114,879	1.0		251	145,546	1.0	
≥25					113	99,976	1.04	0.81–1.34	344	146,054	1.38	1.17–1.63
missing					2	51,375			4	42,689		
Weight change (lbs) (baseline to 10-year follow up)												
<10					167	138,882	1.0		345	183,392	1.0	
≥10 gain					78	70,971	0.94	0.71-1.23	233	100,302	1.36	1.14-1.61
missing					10	56,377			21	50,594		

^a^Adjusted for history of benign breast disease and family history of breast cancer in a first-degree relative; age was the time metric and the model was stratified by age at baseline. ^b^Adjusted for nulliparity and age at first full-term pregnancy, history of benign breast disease, family history of breast cancer in a first-degree relative, average alcohol consumption in the year prior to baseline, and neighborhood socioeconomic status; age was the time metric and the model was stratified by age at baseline. ^c^Adjusted for age at menarche, nulliparity and age at first full-term pregnancy, history of benign breast disease, family history of breast cancer in a first-degree relative, and consumption of a plant-based diet; age was the time metric and the model was stratified by age at baseline. *HT* hormone therapy, *BMI* body mass index

For each menopausal/HT group, we evaluated the association between ER+ breast cancer risk and the full spectrum of body size phenotypes (n = 16), defined by height, adolescent BMI, baseline BMI, weight change between age 18 years and age on joining the cohort, and abdominal adiposity (Table 3). Among premenopausal women, elevated HRs were observed among tall women who experienced weight gain between age 18 and baseline, but whose adult BMI remained within the normal range (HR = 2.02, 95% CI 1.09–3.75) and short women who at baseline were overweight/obese and who developed abdominal adiposity (HR = 2.47, 95% CI 0.94–6.53), although the latter estimate was not statistically significant. The latter estimate also did not significantly differ from that of similar women with gluteal adiposity (HR = 1.88, 95% CI 0.25–13.94), although both phenotypes were characterized by a very small number of cases.

**Table 3:** Full models reflecting body size over the life-course and estrogen-receptor-positive breast cancer risk, by menopausal status and HT use

					Premenopaual			Postmenopausal, current HT use			Postmenopausal, not using HT		
Height	BMI (age 18 years)	BMI (baseline)	Weight change (age 18 years to baseline)	Adiposity	Cases	HR^a^	95% CI	Cases	HR^b^	95% CI	Cases	HR^c^	95% CI
Short	Normal	Normal	Loss		23	1.0		54	1.21	0.89–1.64	18	1.10	0.65 – 1.86
			Stable					172	1.0		65	1.0	
			Gain		6	1.19	0.48 – 2.93	251	1.01	0.83–1.22	115	1.39	1.02 – 1.88
		Overweight/obese		Abdominal	5	2.47	0.94 – 6.53	127	1.15	0.92 – 1.45	125	1.48	1.10 – 2.00
				Gluteal	1	1.88	0.25 – 13.94	110	1.01	0.80 – 1.29	30	2.75	1.78 – 4.25
	Overweight/obese	Normal			39	0.87	0.52 – 1.45	35	1.03	0.71 – 1.48	36	2.63	1.75 – 3.95
		Overweight/obese		Abdominal	13	0.84	0.43 – 1.66	14	0.83	0.48 – 1.43	16	1.34	0.77 – 2.32
				Gluteal	4	1.09	0.38 – 3.15	7	0.83	0.39 – 1.77	1	1.46	0.20 – 10.56
Tall	Normal	Normal	Loss		40	1.21	0.73 – 2.03	19	1.22	0.76 – 1.97	25	1.42	0.90 – 2.26
			Stable					38	0.96	0.67 – 1.36	70	1.36	0.97 – 1.91
			Gain		18	2.02	1.09 – 3.75	117	1.22	0.96 – 1.54	159	1.66	1.24 – 2.21
		Overweight/obese		Abdominal	3	1.07	0.32 – 3.59	39	1.69	1.19 – 2.39	147	2.04	1.52 – 2.73
				Gluteal	2	1.44	0.34 – 6.14	52	1.41	1.03 – 1.92	33	2.09	1.37 – 3.18
	Overweight/obese	Normal			42	0.86	0.52 – 1.43	13	1.12	0.64 – 1.97	22	1.45	0.89 – 2.35
		Overweight/obese		Abdominal	11	0.62	0.30 – 1.28	5	1.06	0.43 – 2.58	20	1.65	1.00 – 2.72
				Gluteal	6	0.91	0.37 – 2.23	2	0.80	0.20 – 3.21	3	1.85	0.58 – 5.90

Body size cut-points: height <65, ≥65 (for premenopausal women and postmenopausal women not using hormone therapy (HT)) and <67, ≥67 (for postmenopausal women currently using HT); Body mass index (BMI) (age 18 years) <20, ≥20 (for premenopausal women) and <25, ≥25 (for postmenopausal women); BMI (baseline) <25, ≥25; weight change loss or gain <25 lbs (stable), gain ≥25 lbs (for premenopausal women) and loss ≥10 lbs (loss), loss <10 lbs or gain <10 lbs (stable), gain ≥10 lbs (for postmenopausal women); adiposity ≥0.50 (abdominal), <0.50 (gluteal) (for premenopausal women and postmenopausal women not using HT) and ≥0.56 (abdominal), <0.56 (gluteal) (for postmenopausal women currently using HT). ^a^Adjusted for history of benign breast disease and family history of breast cancer in a first-degree relative; age was the time metric and the model was stratified by age at baseline. ^b^Adjusted for nulliparity and age at first full-term pregnancy, history of benign breast disease, family history of breast cancer in a first-degree relative, average alcohol consumption in the year prior to baseline, and neighborhood socioeconomic status; age was the time metric and the model was stratified by age at baseline. ^c^Adjusted for age at menarche, nulliparity and age at first full-term pregnancy, history of benign breast disease, family history of breast cancer in a first-degree relative, and consumption of a plant-based diet; age was the time metric and the model was stratified by age at baseline

Among postmenopausal HT users, taller women who had become overweight or obese during adulthood were at increased risk of breast cancer. The difference in the magnitude of risk for those women with abdominal (HR = 1.69, 95% CI 1.19–2.39) vs gluteal (HR = 1.41, 95% CI 1.03–1.92) fat distribution was not statistically significant (*p* = 0.32). Among postmenopausal women not using HT, weight gain since adolescence, among women maintaining a normal BMI, increased risk among both short (HR = 1.39, 95% CI 1.02–1.88) and tall (HR = 1.66, 95% CI 1.24- 2.21) women. Short women with gluteal adiposity at baseline (HR = 2.75, 95% CI 1.78–4.25) were at greater risk than those with abdominal adiposity (HR = 1.48, 95% CI 1.10–2.00) (*p* = 0.003); the location of body fat among tall women did not impact risk, although being overweight/obese did (HR = 2.09, 95% CI 1.37–3.18 and HR = 2.04, 95% CI 1.52–2.73 for gluteal and abdominal adiposity, respectively). Among short women who were overweight/obese in adolescence, those with normal BMI at baseline were at increased risk (HR = 2.63, 95% CI 1.75–3.95), whereas those who continued to be overweight/obese with greater abdominal girth were not (HR = 1.34, 95% CI 0.77–2.32) (*p* = 0.03).

To obtain the most parsimonious predictive models, rare phenotypic combinations (i.e., those with <10 cases) were collapsed into single groups, as were groups with similar risk estimates, using an iterative process. In premenopausal women, only adolescent (age 18 years) BMI remained predictive of risk (see Table 2). Final models for postmenopausal women are presented in Table 4. Among HT users, relative to short women, tall overweight/obese women were at increased risk of breast cancer (HR = 1.36, 95% CI 1.13–1.64), but tall women of normal weight were not (HR = 1.11, 95% CI 0.95–1.30). Among women not using HT and who had maintained a normal weight throughout life, the effect of height approached statistical significance (HR = 1.35 95% CI 1.00–1.81). Shorter women who had been overweight/obese since adolescence were not at increased risk (HR = 1.33, 95% CI 0.84–2.10) whereas taller women were (HR = 1.49, 95% CI 1.06–2.10); although these HRs did not differ statistically. Adult weight gain was also associated with increased risk (HR = 1.36, 95% CI 1.03–1.81 among shorter women and HR = 1.77, 95% CI 1.40–2.25 among taller women). Among shorter women gluteal adiposity was associated with a greater increase in risk (HR = 2.70, 95% CI 1.77–4.10) than was abdominal adiposity (HR = 1.45, 95% CI 1.10–1.92) (*p* = 0.003).

**Table 4:** Best fitting models describing the association between life-course body size and estrogen-receptor-positive postmenopausal breast cancer risk, by HT use

Height	BMI (age 18 years)	BMI (baseline)	Weight change (age 18 years to baseline)	Adiposity	Cases	HR	95% CI
Current HT use							
Short					887	1.0^b^	
Tall		Normal			190	1.11	0.95–1.30
		Overweight/obese			130	1.36	1.13–1.64
Not using HT							
Short	Normal	Normal	<10 lbs		83	1.0^c^	
			≥10 lbs		115	1.36	1.03–1.81
		Overweight/obese		Abdominal	125	1.45	1.10–1.92
				Gluteal	30	2.70	1.77–4.10
	Overweight/obese	Normal			36	2.57	1.74–3.81
		Overweight/obese			24	1.33	0.84–2.10
Tall	Normal		<10 lbs		95	1.35	1.00–1.81
			≥10 lbs		384	1.77	1.40–2.25
	Overweight/obese				54	1.49	1.06–2.10

Body size cut-points: height <65, ≥65 (for postmenopausal women not using hormone therapy (HT)) and <67, ≥67 (for postmenopausal women currently using HT); body mass index (BMI) (age 18 years) <25, ≥25; BMI (baseline) <25, ≥25; adiposity ≥0.50 (abdominal), <0.50 (gluteal). ^a^Adjusted for history of benign breast disease and family history of breast cancer in a first-degree relative; age was the time metric and the model was stratified by age at baseline. ^b^Adjusted for nulliparity and age at first full-term pregnancy, history of benign breast disease, family history of breast cancer in a first-degree relative, average alcohol consumption in the year prior to baseline, and neighborhood socioeconomic status; age was the time metric and the model was stratified by age at baseline. ^c^Adjusted for age at menarche, nulliparity and age at first full-term pregnancy, history of benign breast disease, family history of breast cancer in a first-degree relative, and consumption of a plant-based diet; age was the time metric and the model was stratified by age at baseline

Finally, we evaluated whether later adult body size (i.e., BMI at the 10-year follow up and weight change between baseline and the 10-year follow up) modified any of the risk estimates in Table 4 (data not shown). We had too few premenopausal women to conduct this additional analysis. Among postmenopausal HT users, no modification was observed. Among shorter postmenopausal women not using HT who had gained weight between age 18 years and baseline (HR = 1.36, 95% CI 1.03–1.81 (Table 4)), additional weight gain of 10 1bs or more later in life was associated with a substantial increase in risk (HR = 2.57, 95% CI 1.60–4.12), whereas risk was not increased among those who did not gain the additional weight (HR = 1.23, 95% 0.79– − 1.91). Later adult body size did not modify any of the other observed associations with postmenopausal breast cancer.

## Discussion

Our analysis suggests that the relationships between body size phenotypes, based on measures from adolescence through adulthood, and ER+ breast cancer are complex. We observed that the risk of premenopausal breast cancer was influenced by adolescent, but not adult, body size, with greater body mass associated with a reduction in risk. In addition, while becoming overweight/obese in adulthood impacted the risk of postmenopausal breast cancer, having been overweight/obese over life since adolescence did not. The use of HT substantially increases breast cancer risk [16, 17]; this risk was increased further among those with the largest body stature (i.e., those who were tall and overweight/obese).

Among postmenopausal women not using HT, the relationship between body size and risk was complex, with the largest effects of adiposity observed among short women. Among short women, while weight gain during adulthood resulting in abdominal adiposity increased risk, gluteal adiposity was significantly more detrimental; however, among tall women, body fat distribution did not modify the risk associated with being overweight or obese in adulthood. Among short women, continued weight gain throughout adulthood was also associated with a significant increase in risk. Height was associated with increased risk, with borderline statistical significance.

Our findings on the associations between single body-size measures and breast cancer risk (Table 2) were consistent with the general consensus in the literature [1]. However, our life-course approach also suggests that among postmenopausal women currently using HT, the previously observed overall increased risk associated with greater height may in fact be limited to taller women who also have greater overall body mass. In addition, among postmenopausal women not using HT, the overall increased risk associated with abdominal adiposity was not observed when height and adolescent body mass were taken into account. This finding is consistent with the literature review published by Pischon et al. [7]. In fact, among short postmenopausal women who became overweight or obese due to adult weight gain after age 18 years but prior to baseline, risk associated with gluteal adiposity was substantially greater than that due to abdominal adiposity. This finding is consistent with the recent report by Harding et al. [18] suggesting that hip circumference may be a better predictor of postmenopausal breast cancer than waist circumference.

Apart from single measures assessed at different ages and reported separately, there is little literature on the lifetime effects of body size on breast cancer risk. Studies in France and Mexico have looked at body shape trajectories from the prepubertal period to age 35 or 40 years based on Sørensen’s pictograms [19, 20]. In the French study, relative to women reporting the leanest body shapes throughout life, women who consistently reported mid-range or the largest body shapes were at reduced risk of ER+ progesterone-receptor-positive (PR+) postmenopausal breast cancer; women reporting greater body size during adolescence than during adulthood were at similar risk but the effect estimate was not significantly significant [20]. The authors concluded that adiposity at puberty reduced the risk of later-life breast cancer and this effect was independent of later-life body size [20]. However, the opposite was observed in the Mexican study: women consistently reporting mid-range or the largest body shapes and those reporting a moderate increase in body shape size over time were at increased risk of postmenopausal breast cancer [19]. Neither study found statistically significant associations with premenopausal breast cancer.

In a similar analysis, conducted in the USA, assessing trajectories from age 5 to 60 years, moderate or marked increases in body size increased the risk of postmenopausal breast cancer, but there was no increased risk among those whose body size was relatively stable throughout life, regardless of their absolute body size [21]. While these findings are not directly comparable to ours, the increased risk associated with adiposity acquired during adulthood in the studies from Mexico (in which less than 25% of women had ever used HT) and the USA (in which no heterogeneity by HT use was reported) is generally consistent with the patterns we observeS in postmenopausal women who were not using HT.

Unlike adult-acquired body mass, several previous studies have found that being overweight/obese over lifetime (i.e., since adolescence) may not impact the risk of postmenopausal breast cancer [4, 9, 10, 20] or in some cases, may even reduce a woman’s risk [20]. Overall our findings are consistent with this observation. The implication of these findings for more recent generations, however, is unclear as the relative contribution of heredity, overeating, sedentary behavior, and the altered hormone levels associated with each, to adolescent obesity may differ in these older cohorts than in future ones. Despite our overall findings on adolescent obesity, we did observe a significant increase in risk limited to short women who were overweight/obese in adolescence but of normal adult body mass. The reason for this exception is not clear and may be due to misclassification or chance.

Mechanisms related to sex steroid hormones, inflammation, and glucose/insulin are suggested as the pathways most likely to be involved in anthropometric-related carcinogenesis [1, 2, 7]. Adult height in women is usually established by mid-adolescence and is a marker of aggregated fetal and childhood factors promoting linear growth, including early-life nutrition and environmental exposures, hormone profiles and the rate of sexual maturation, and genetics [1, 2] [22]. In premenopausal women the female sex steroid hormones are produced largely by the ovaries, whereas in postmenopausal women estrogens are largely the result of the conversion of adrenal androgens to estrogens in adipose tissue. Adolescent obesity is often associated with earlier menarche and irregular and anovulatory menstrual cycles, resulting in altered levels of ovarian hormones, sex hormone binding globulin, and insulin-like growth factor-1 [2, 6, 20]. These alterations in turn may result in earlier mammary cell differentiation and lifelong reduction in mammary cell growth and proliferation reducing the risk of breast cancer later in life [2, 13, 20].

Weight gain is an indicator of sustained positive energy balance and, along with adult obesity, affects circulating hormones, growth factors, insulin, and inflammatory cytokines which together can increase carcinogenesis, decrease apoptosis, and stimulate low-grade chronic inflammatory responses [1, 2, 7, 19]. Adult weight gain primarily reflects the disposition of fat mass with abdominal fat associated with impaired glucose metabolism, increased insulin resistance, and in postmenopausal women, altered estrogen synthesis [1, 6, 7, 13, 23, 24]. Abdominal adiposity is more closely correlated with visceral adipose tissue, which is more metabolically active, secreting more cytokines and hormones, than subcutaneous adipose tissue [7, 24]. Gluteal adipocytes, on the other hand, are more likely to be responsive to female sex steroid hormones [25]. Finally, exogenous HT may obscure the effect of adiposity on breast cancer risk by artificially increasing the levels of circulating estrogens [6, 23].

Several limitations of the present analysis should be noted. First, while determined *a priori*, our approach to and interpretation of the analysis is somewhat agnostic, has a certain degree of subjectivity, involves multiple comparisons, and for some subgroups is based on small numbers of cases. However, we used quantitative methods, i.e., the Wald test, to determine whether specific subgroups differed. While collapsing subgroups based on small numbers of cases may have masked some associations, this may also have reduced over-interpretation of erroneous patterns.

Second, we included only 16 body-size phenotypes in our analysis. Examining additional phenotypes, such as absolute weight gain among women with who were overweight or obese in adulthood, being overweight or obese over life, or body fat distribution among women of normal BMI, may be possible in larger consortial datasets.

Third, our focus was on body size over the life-course but the available data limited us to evaluation at several specific points in time only, i.e., age 18 years, baseline (generally reflecting ages 34–46 in premenopausal women, 52–65 in postmenopausal women using HT, and 55–72 in postmenopausal women not using HT), and 10 years after baseline, and the changes between these points.

Fourth, while data on menopausal status and HT use were updated, this was only done at 5-year and 10-year follow up, rather than more often, and thus, has some built-in imprecision. The largest change in HT use over the follow-up period, however, was the substantial population cessation of HT use following the publicity surrounding the findings of the Women’s Health Initiative in 2002–2003 [16, 17]. This event fell between our two follow-up questionnaires, thus, women who quit use as a result of this event contributed person-time and events to the current HT group for a few “extra” years as opposed to being switched to the “not currently using HT” group immediately, which functionally precluded an assumption of instantaneous risk reduction. Relatedly, some misclassification may have been introduced into the analysis of adult (baseline) BMI and WHtR in women who were premenopausal at baseline but postmenopausal at the 5-year follow up. They would have contributed person-time to one of the postmenopausal subgroups between the 5-year and 10-year follow up, but unlike women who were postmenopausal at baseline or became postmenopausal after the 5-year follow up, their adult BMI and WHtR data would have reflected a premenopausal assessment rather than a postmenopausal one during the 5 years of the follow-up period. However, these women accounted for only 10% and 15% of those contributing to the analyses of postmenopausal/current HT and postmenopausal/not using HT groups, respectively. After the 10-year reassessment of both menopausal status and body size, this would not have been a concern.

Fifth, the anthropometric data used in the present analysis were self-reported, which can result in measurement error due to lack of knowledge, most notably for weight at age 18 years, or desire to report a socially more normative value. However, our validation study suggested excellent reproducibility and validity, minimizing such concerns [4]. Last, we did not include other body size measures, such as weight cycling, which in one study was found to be associated with the greatest risk of breast cancer [5].

Notable strengths of this analysis include the unique conceptual approach taken to assessing the effects of body size over the life-course and the conduct of these analyses in a large cohort of women who had detailed data on anthropometry and breast cancer outcomes over the course of more than 15 years. We included both dynamic (e.g., weight change) and static measures (e.g., BMI) of body size, of which the former may better reflect age-related metabolic changes, while the latter reflects the effects of absolute size [6]. In addition, when assessing abdominal (android) versus gluteal (gynoid) obesity, we used the ratio of waist circumference to height rather than the more common waist circumference or waist-to-hip ratio, as the former is a better predictor of visceral fat [12].

## Conclusions

Taking into account absolute body mass, changes in adiposity over time, and body fat distribution, we identified lifetime (since adolescence) obesity phenotypes which were associated with breast cancer risk in our large cohort. These findings may aid in improving risk prediction models and developing targeted interventions, and may clarify inconsistent findings across studies to the extent that the composition of the study populations differ in regards to important anthropometric indicators. To the extent that equally detailed anthropometry is available in other studies, similar analyses conducted in large consortial datasets, such as the Cohort Consortium or the Harvard Diet Pooling Project, are needed to confirm our findings and improve statistical power for evaluating effect modification across rare phenotypes.

## Abbreviations

BMI: Body mass index
CCR: California Cancer Registry
CI: Confidence interval
CTS: California Teachers Study
ER+: Estrogen receptor-positive
HR: Hazard rate ratio
HT: Hormone therapy
IQR: Interquartile range
PR+: Progesterone receptor-positive
WHtR: Waist-to-height ratio
